# *Osteopathic Medicine and Primary Care: *a new journal for changing times

**DOI:** 10.1186/1750-4732-1-1

**Published:** 2007-01-12

**Authors:** John C Licciardone, Roberto Cardarelli

**Affiliations:** 1 Osteopathic Research Center, University of North Texas Health Science Center-Texas College of Osteopathic Medicine, Fort Worth, TX, USA; 2 Department of Family Medicine, University of North Texas Health Science Center-Texas College of Osteopathic Medicine, Fort Worth, TX, USA

## Abstract

*Osteopathic Medicine and Primary Care *is dedicated to the rapid and universal dissemination of peer-reviewed research and scholarly work within its scope. It aims to bridge diverse professional communities by providing a common forum for the publication of research relevant to the clinical practice of primary care.

## *Osteopathic Medicine and Primary Care*

*Osteopathic Medicine and Primary Care *is dedicated to the rapid and universal dissemination of peer-reviewed research and scholarly work related to osteopathic and allopathic clinical practice. The scope of primary care covered by the journal includes all aspects of family medicine, internal medicine, pediatrics, and obstetrics and gynecology. Relevant topics include therapeutics, diagnostic tests, preventive services, clinician-patient interactions, and ethics, just to name a few. Research that is unique to primary care responds to the need for evidence-based findings that can be generalized and applied to populations that are served by primary care clinicians. Such research is integral to sustaining the primary care community [1], particularly with threats to primary care as a viable specialty [2]. Practice-based research networks (PBRNs), which include nationally or geographically defined groups of clinics that collaborate to conduct studies, are also encouraged to submit manuscripts. Research conducted by PBRNs has the potential to impact the treatment of ailments that are seen in everyday practice [3], such as the use of antibiotics for upper respiratory infections [4]. The journal also welcomes submissions involving health services research or public health research that deals with primary care issues. Thus, manuscripts may range from those that involve individual patients to others that address entire populations.

Naturally, *Osteopathic Medicine and Primary Care *also seeks submissions on topics that are uniquely osteopathic. Osteopathic medicine in the United States still struggles to establish a unique professional identify [5]. It also faces professional uncertainty as many osteopathic medical school graduates continue to enter allopathic graduate medical education programs in primary care [6]. Although systematic review and evidence-based assessment of osteopathic manipulative treatment (OMT) has recently surfaced [7], much more research is needed to contribute to the development of clinical practice guidelines and public policy relative to OMT. While the aforementioned challenges are particularly germane to American osteopathic medicine, it must be recognized that the international community may be more familiar with "osteopathy," as practiced in Europe or Australasia. Consequently, the journal welcomes submissions from all corners of the world. Relevant journal topics may include, but are not limited to, the anatomical and physiological bases of OMT, somatic dysfunction, reliability of osteopathic palpatory findings, mechanisms of action of OMT techniques, efficacy and clinical outcomes associated with OMT, utilization of osteopathic health services, osteopathic physician-patient interactions, and osteopathic medical education.

## Open access publishing

As a BioMed Central independent, open access journal, *Osteopathic Medicine and Primary Care *is strategically placed and poised to assume a leading role in disseminating peer-reviewed research and scholarly work within its scope. The journal includes an Editorial Board [8] of diverse professional orientation that will continue to grow in numbers and geographical sphere over time. There are numerous reasons for publishing in the journal. First, all articles are open access (i.e., they are universally accessible online without charge). Second, upon acceptance, all articles are immediately published and then indexed in PubMed. They are also deposited and permanently archived in PubMed Central, the full-text repository of life science literature maintained by the United States National Library of Medicine. Third, authors retain the copyright to their articles and grant any third party the right to reproduce and disseminate the article, provided that it is cited correctly. This facilitates the circulation of articles via print copies, e-mail distribution, and posting on the Web. Fourth, online publishing provides unlimited space for appending tables, extensive data, figures, photographs, and video footage to an article. The advantages of open access publishing are likely to accelerate recognition and dissemination of research findings, as indicated by a recent bibliometric comparison of open access and non-open access articles published in the same journal (*Proceedings of the National Academy of Sciences*) [9]. This study, which controlled for several potential confounders, found that open access articles were more likely to be cited than non-open access articles in the first 4–10 months after publication (odds ratio, 2.1; 95% confidence interval, 1.5–2.9), and that the citation advantage of open access articles increased in the 10–16 months after publication (odds ratio, 2.9; 95% confidence interval, 1.5–5.5).

All manuscripts are submitted to *Osteopathic Medicine and Primary Care *and then reviewed using an electronic system (accessible online [10]) that optimizes correspondence with the journal's Editorial Team and thereby facilitates timely reviews and editorial decisions. Authors are required to nominate at least 3 potential reviewers at the time of submission, although the journal is not obligated to select any of the suggested reviewers. An initial screening is performed by the Editorial Team to ensure that a submission falls within the scope of the journal and that basic quality standards are met. At least 2 reviewers and, if necessary, a biostatistical consultant are assigned to each acceptable submission. Author information is provided to reviewers; however, authors are blind to reviewer identities. The journal's goal is to have a time lag of no more than 2–6 months from manuscript submission to publication. Generally, the time lag will depend upon the quality of the original submission and the rapidity with which requested manuscript revisions are provided by the authors.

In the absence of revenue from subscriptions, article processing charges (APCs) cover the cost of the journal's daily operations, rapid and universal dissemination of its contents, and indexing and permanent archiving of its articles. Authors at institutions that are members of BioMed Central will receive either a full or partial discount of the APCs. Many funding agencies, such as the United States National Institutes of Health, explicitly authorize the use of grant monies for payment of APCs to encourage open access publication. The corresponding author must confirm, on submission, that the APC will be paid should the article be accepted for publication in *Osteopathic Medicine and Primary Care*. If a manuscript is ready to be accepted, following peer review, the corresponding author will be notified that the APC payment is due. Additional information about APCs is available online [11].

## Conclusion

*Osteopathic Medicine and Primary Care *provides the opportunity to publish high-impact research with rapid and universal dissemination. We are eager to receive and review the work of investigators and scholars in the diverse fields related to primary care.

## Competing interests

JCL and RC are Editor-in-Chief and Associate Editor of *Osteopathic Medicine and Primary Care *respectively.
